# Modified activities of macrophages’ deubiquitinating enzymes after *Francisella* infection

**DOI:** 10.3389/fimmu.2023.1252827

**Published:** 2023-09-29

**Authors:** Vera Vozandychova, Pavel Rehulka, Kamil Hercik, Petra Spidlova, Pavla Pavlik, Jaroslav Hanus, Romana Hadravova, Jiri Stulik

**Affiliations:** ^1^ Department of Molecular Pathology and Biology, Faculty of Military Health Sciences, University of Defence, Hradec Kralove, Czechia; ^2^ Institute of Organic Chemistry and Biochemistry, Academy of Sciences of the Czech Republic, Prague, Czechia; ^3^ Department of Chemical Engineering, University of Chemistry and Technology, Prague, Czechia

**Keywords:** *Francisella*, deubiquitination, DUBs, exosomes, extracellular vesicles, USP10, UCH-L5, USP25

## Abstract

*Francisella tularensis* influences several host molecular/signaling pathways during infection. Ubiquitination and deubiquitination are among the most important regulatory mechanisms and respectively occur through attachment or removal of the ubiquitin molecule. The process is necessary not only to mark molecules for degradation, but also, for example, to the activation of signaling pathways leading to pro-inflammatory host response. Many intracellular pathogens, including *Francisella tularensis*, have evolved mechanisms of modifying such host immune responses to escape degradation. Here, we describe that *F. tularensis* interferes with the host’s ubiquitination system. We show increased total activity of deubiquitinating enzymes (DUBs) in human macrophages after infection, while confirm reduced enzymatic activities of two specific DUBs (USP10 and UCH-L5), and demonstrate increased activity of USP25. We further reveal the enrichment of these three enzymes in exosomes derived from *F. tularensis*-infected cells. The obtained results show the regulatory effect on ubiquitination mechanism in macrophages during *F. tularensis* infection.

## 1 Introduction

*Francisella tularensis*, an intracellular bacterium, is one of the most virulent pathogens classified under Category A as a Potential Bioterrorism Agent. The pathogen causes tularemia, a zoonotic disease for which there is currently no licensed vaccine available and that generally is treated by antibiotics (1). *F. tularensis* has the ability to infect many different hosts, such as mammals, fish, amphibians, protozoa and arthropods. A human can be infected by contact with an infected animal, by ingesting contaminated food or water, by inhaling a contaminated aerosol, or by vector arthropods (2). The manifestation of the disease in humans differs depending on the route of infection, and therefore it is distinguish the ulceroglandular, oculoglandular, pulmonary or gastrointestinal form. Manifestations of tularemia include non-specific flu-like symptoms or ulcers, conjunctivitis, a dry cough with shortness of breath or symptoms of a gastrointestinal infection (3). During infection, *F. tularensis* primarily infects macrophages, but there is evidence that it can also invade other cell types, such as neutrophils, dendritic cells, and hepatocytes (4). After phagocytosis, the bacteria reside within the phagosome of the macrophage. They can escape and proliferate in the cytosol of the host cell, eventually inducing apoptosis and beginning a new infectious cycle in the other host cells (5). The escaping step can be observed within the first to fourth hour after infection of host cells (5, 6).

Ubiquitin (Ub) is a small, conservative protein consisting of 76 amino acids that performs an essential role in post-translational modification of proteins. The process, known as ubiquitination, involves three distinct steps. First, an ATP-dependent Ub-activating enzyme (E1) activates the C-terminus of the Ub molecule. Next, E1 is replaced by a Ub-conjugating enzyme (E2), which is then followed by a Ub-ligase (E3). E3 ligase is responsible for the specificity of the Ub-substrate bond, which occurs when Ub binds to lysine residues on the substrate via its C-terminus (7). Ubiquitination, a protein modification involving the attachment of ubiquitin molecules, has a profound effect on protein localization and endocytosis. Different types of polyubiquitin chains can be generated through polyubiquitination, with ubiquitin molecules linking via lysine residues K6, K11, K27, K29, K33, K48, and K63. Best described are the K48- and K63-linked polyubiquitin chains. Chains involving K48 are targeted for proteasomal degradation, while those involving K63 have a regulatory role in several cell processes, such as DNA repair, signal transduction, endocytosis, vesicular trafficking, and cell cycle progression (8, 9).

Deubiquitinating enzymes (DUBs) are proteases that cleave ubiquitin chains, thereby playing a role in maintaining ubiquitin homeostasis and modifying Ub-protein conjugates. The activity of the host ubiquitin system and, in particular, DUBs during an infectious process can be modulated by various pathogens (10). *F. tularensis* live vaccine strain (LVS) has been found to interfere with K63 polyubiquitination, thereby modulating and inhibiting TRAF6 and TRAF3 complexes that control pattern recognition receptor response (11). *F. tularensis* requires USP22, the host ubiquitin-specific hydrolase, and CDC27, a protein responsible for ubiquitin-mediated proteolysis of B-type cyclins, for its proliferation within the cytosol of mammalian cells, suggesting that it exploits the host ubiquitination system (12). Study of *Francisella novicida* has suggested that E3 ligase HECTD3, which promotes K63-linked polyubiquitination of TRAF3 and thus type I interferon production, is affected by bacterium during infection (13).

Exosomes are small, endosomal membrane microvesicles released by all eukaryotic cells. Most commonly, their sizes range from roughly 30 nm to 150 nm. Their content and function depend on the given situation and the type of cells by which they are produced, and at the same time reflect their metabolic state (14). Their biologically active content can offer prognostic information on a wide range of diseases (15–17). Recent studies have shown that exosomes, released from cancer cells, play major role in tumor-related processes (18). The roles of exosomes in inflammasome-dependent immune response are discussed by 19.

In the work presented here, we focused on DUB enzyme activity during infection of human THP-1 macrophages by *F. tularensis* subsp. *holarctica* FSC200. We revealed that *F. tularensis* interferes with the host’s ubiquitination system. We observed increased activity of deubiquitinating enzymes in THP-1 cells. Using proteomic analysis of selectively isolated enzymatically active DUBs, we confirmed decreased amounts of active USP10 and UCH-L5, as well as increased amount of active USP25, in cells after infection. We also detected enrichment of these three enzymes in THP-1-derived exosomes after *F. tularensis* infection. Our findings shed light on the molecular mechanisms of this pathogenic bacterium and contribute to better understanding the role of DUBs during infection. The alteration of the activities of particular DUBs may play an important role in *Francisella’*s ability to resist and escape the host defense mechanisms, and therefore this study could contribute to the development of an effective tularemia treatment.

## 2 Materials and methods

### 2.1 Cells and cultivation

Human THP-1 monocytes (ATCC TIB-202) were cultivated in RPMI 1640 medium without glucose (Gibco, 11879020) supplemented with 10% fetal bovine serum (Gibco, 16140071), 2 mM glutamine (Gibco, 25030081), and 100 U/mL penicillin–streptomycin (Gibco, 15140122) at 37°C in atmosphere of 5% CO_2_. The cell lines presented in this study were obtained from Sigma-Aldrich (88081201). To induce THP-1 cells differentiation to macrophages, 100 nM phorbol-12-myristate 13-acetate (PMA; Sigma-Aldrich, P1585) was used.

Gram-negative bacterium *Francisella tularensis* subsp. *holarctica* FSC200 was grown in brain heart infusion medium (Becton Dickinson, 211059) supplemented with 0.1% cysteine (Fluka, 30120) at 37°C (200 rpm) or on McLeod agar plates at 37°C in normal atmosphere. *F. tularensis* subsp. *holarctica* FSC200 is classified as a pathogen of Biosafety Level 2 (BSL-2) according to the Regulation of the Government of the Czech Republic and thus all experiments were performed under the BSL-2 conditions.

### 2.2 Infection of THP-1 cells

Submerged cultivated THP-1 monocytes were plated onto dishes at concentration of 10^8^ cells/dish (17,571 mm^2^). Following 3 days of cell differentiation by 100 nM phorbol 12-myristate 13-acetate (PMA), the morphology of the differenciated THP-1 macrophages was validated using microscopy (data not shown). THP-1 macrophages were infected by *F. tularensis* subsp. *holarctica* FSC200 strain at 500:1 multiplicity of infection. Cells were incubated for 10 or 60 min at 37°C. Uninfected cells were used as a negative control. At the proper time point, THP-1 cells were washed once with ice-cold phosphate-buffered saline (PBS, Gibco, 10010056). Infected and uninfected cells were scraped into ice-cold PBS, centrifuged for 5 min at 500 × g, and cell pellets were used for further analysis.

### 2.3 LDH cytotoxicity assay

The cell culture supernatant from uninfected and infected macrophages at time points 10 and 60 min were analyzed by LDH Cytotoxicity Assay (Pierce, 88954). The measurement of cytotoxicity mediated by infection was in accordance with the manufacturer’s protocol.

### 2.4 Cell lysis

THP-1 macrophage pellets were lysed by French pressure cell press in lysis buffer (150 mM NaCl, 5 mM MgCl_2_, 50 mM Tris pH 7.5, 1% glycerol, 2 mM 2-mercaptoethanol,1 mM phenylmethanesulfonyl fluoride [Roche, 10837091001], 2 U/mL Turbo™ DNase [Invitrogen, AM2238], and 20 U/mL RNase A/T1 mix [Thermo Scientific, EN0551]). The cell lysates were centrifuged for 15 min at 21,000 × g at 4°C to remove cell debris. The protein concentration of the supernatant was determined using the Pierce™ BCA Protein Assay Kit (Pierce, 23225) or Qubit Protein Assay Kit (Invitrogen, Q33212) according to the manufacturer’s protocol.

### 2.5 Deubiquitinase assay

The cell lysates of uninfected and infected macrophages at time points 10 and 60 min were analyzed by deubiquitinase assay kit (Abcam, ab241002). The deubiquitinase activity was measured utilizing a fluorescent deubiquitinase substrate according to the manufacturer’s protocol.

### 2.6 Activity-based probe reaction and immunoprecipitation

Samples containing equal amounts (1 mg) of cell lysates were subjected to enzymatic reaction with the ubiquitin propargylamid (HA-Ahx-Ahx-Ub-PA; UbiQ, UbiQ-078) or vinylmethyl ester (HA-Ahx-Ahx-Ub-VME; UbiQ, UbiQ-035) HA-tagged probe at a probe:protein ratio of 1:250 for 30 min at 37°C. After the enzymatic reaction, the samples were immunoprecipitated using 100 µL of anti-HA resin (Millipore, E6779) for 2 h at 4°C. The resin was washed once with 3 volumes of lysis buffer; then once with 3 volumes of 300 mM NaCl, 50 mM Tris pH 7.5, 2 mM 2-mercaptoethanol; and finally three times with 3 volumes of 150 mM NaCl, 50 mM Tris pH 7.5, and 2 mM 2-mercaptoethanol. Elution was performed twice with 100 µL of 50 mM NaOH. The pH of the eluate was adjusted with 1 M Tris to pH 7.5.

### 2.7 Proteomic analysis

#### 2.7.1 TMT6 isobaric labeling

Following the manufacturer’s protocol, 100 µg of proteins isolated from cell lysates were prepared for TMT labeling using the TMTsixplex Isobaric Mass Tagging kit (Thermo Fisher, 90064). Proteins were digested overnight at 37°C with trypsin (SOLu-trypsin dimethylated; Sigma-Aldrich, EMS0005) in the ratio trypsin:proteins = 1:30 (w/w). Upon acidification of samples with 1% trifluoroacetic acid (TFA), the sodium deoxycholate (DOC; Sigma-Aldrich, D5670) precipitate was formed and then removed by centrifugation for 1 min 3,000 × g. An equal volume of ethyl acetate was added to the supernatant and the mixture was vortexed and centrifuged for 1 min at 3,000 × g. The upper organic phase was removed, and the extraction process was repeated four times to remove DOC. The samples were vacuum-dried and redissolved in 71 µL of 100 mM triethyl ammonium bicarbonate and isobaric labeled by TMTsixplex Isobaric Mass Tagging Reagents (Thermo Fisher, 90064). The pooled samples were desalted using Empore C18-SD extraction disk cartridges (4 mm/1 mL; Supelco, 66871-U), eluted by 60% acetonitrile (ACN)/0.1% TFA, vacuum-dried, then stored at −20°C. The isobaric-labeled peptides were fractionated by a home made gradient capillary chromatography wherein reverse phase C18 under basic pH and hydrophilic interaction liquid chromatography conditions were used. Each fraction was vacuum-dried and stored at −20°C.

#### 2.7.2 Label-free quantitation (LFQ) analysis

The eluted proteins were precipitated by 0.18 volume of 100% trichloroacetic acid (Sigma-Aldrich, T4885), incubated for 4 h on ice, then centrifuged for 30 min at 21,000 × g and 4°C. The supernatant was discarded; the pellet was washed with ice-cold 90% acetone and then centrifuged for 15 min at 21,000 × g and 4°C. The acetone was removed and the pellet was dried. Next, the pellet was solubilized in 4% DOC in 25 mM ammonium bicarbonate (ABC; Fluka, 09830). The proteins were reduced with 4 mM dithiothreitol (DTT; Fluka, 43819) in 25 mM ABC and directly alkylated with 16 mM iodoacetamide (IAA; Sigma-Aldrich, I1149) in 25 mM ABC. The excess of IAA reagent was quenched by the addition of DTT to a final concentration of 4 mM DTT in 25 mM ABC. Digestion was performed overnight at 37°C using SOLu-trypsin in a ratio of 1:30 (w/w). Next, 20 µL 1% TFA (Fluka, 34967) was used for acidification the sample mixture. The DOC precipitate was removed by centrifugation and ethyl acetate extraction as described above. The samples were desalted using Empore C18-SD extraction disk cartridges (4 mm/1 mL; Supelco, 66871-U).

#### 2.7.3 Liquid chromatography–mass spectrometry (LC-MS)

The samples were dissolved in 20 µL of 2% ACN/0.1% TFA (v/v), and 1 µL of the sample was analyzed using a gradient nanoLC system with UV detection (UltiMate 3000 HPLC system (Dionex, USA) containing a µ-Precolumn (300 µm × 5 mm, C18PepMap 5 µm 100 Å particles; Dionex) and analytical NanoEase column (100 µm × 150 mm, Atlantis C18 3 µm 100 Å particles; Waters, USA). The optimized sample loadings were then used for LC-MS analysis on the UltiMate 3000 RSLC-nano HPLC system (Dionex) with a trap column (75 µm × 20 mm) packed with 3 µm Acclaim PepMap100 C18 particles and a separation column (75 µm × 150 mm) packed with 2 µm Acclaim PepMap RSLC C18 particles, all coupled to the QExactive system (Thermo Fisher Scientific, USA) in positive ion mode. Instrumental data acquisition was done using Xcalibur software v. 4.2.47 and Tune control software v. 2.11.0.3006. The instrument settings for TMT quantitation analysis were set as follows: full MS scan (400–1650 m/z) at 70,000 full width at half maximum (FWHM) with maximum filling time 100 ms and automatic gain control (AGC) target 3E6; top 10 precursors in MS/MS at 35,000 FWHM with isolation window 1.2 m/z, fixed first mass at 110 m/z, maximum filling time 250 ms, and AGC target 2E5. The settings for label-free quantitation analysis were these: full MS scan (350–1650 m/z) at 70,000 FWHM with maximum filling time 100 ms and AGC target 1E6; top 12 precursors in MS/MS at 17,500 FWHM with isolation window 1.6 m/z, fixed first mass at 140 m/z, maximum filling time 100 ms, and AGC target 1E5.

#### 2.7.4 MS data analysis

The raw spectra files thus obtained were processed in Proteome Discoverer software (Thermo Fisher Scientific, v. 2.4.1.15) to identify proteins and to quantify relative protein abundance in the samples. The data processing workflow contained a spectrum selector, non-fragment filter, top N peaks filter (LFQ only), precursor detector, SequestHT search engine, Percolator validator, Spectrum Confidence Filter, SequestHT search engine and Percolator validator (second round search, TMT quantitation only) nodes. The parameters for the first SequestHT database search were: protein database – UniProt human reference proteome UP000005640 and UniProt *Francisella tularensis* reference proteome UP000006302; enzyme – trypsin; maximum missed cleavage sites – 1; min. peptide length – 7; precursor mass tolerance – 10 ppm; fragment mass tolerance – 0.02 Da; weight of b- and y-ions – 1; static modifications – carbamidomethyl/+57.021 (C); dynamic modifications – oxidation/+15.995 Da (M); dynamic modifications (protein terminus) – acetyl/+42.011 Da (N-terminus), met-loss/−131.040 Da (M), met-loss+acetyl/−89.030 Da (M). The search results thus obtained (msf file) were processed in the consensus workflow containing PSM Grouper, Peptide Validator, Protein and Peptide Filter (two peptides with strict target FDR 0.01), Protein Scorer, Protein FDR Validator, Protein Grouping, Protein in Peptide Annotation, Modification Sites, Protein Annotation and Protein Marker nodes. In the TMT6 labeling experiment, Reporter Ions Quantifier node was used in the processing workflow and the Reporter Ions Quantifier node in the consensus workflow (with pairwise computed protein ratio and background-based *t*-testing). For the label-free quantitation analysis, Minora Feature Detector node was used in the processing workflow and Feature Mapper and Precursor Ions Quantifier (with pairwise computed protein ratio and background-based *t*-testing) nodes were applied in the consensus workflow.

The raw MS data as well as processed identification results have been deposited to the ProteomeXchange Consortium via the PRIDE partner repository with the dataset identifier PXD043492 (DOI: 10.6019/PXD043492).

### 2.8 Immunoblot analysis

Eluates and cell or exosome lysates were separated by SDS-PAGE electrophoresis in gradient 4–12% NuPAGE gels (Invitrogen, NP0321) and transferred onto PVDF membrane (Bio-Rad, 162-0264) using Western blot. Membranes were blocked for 1 h in Blotting-Grade Blocker (Bio-Rad, 165-3301) and subsequently incubated with primary antibodies for 1 h at room temperature. The TBS (tris-buffered saline) buffer supplemented with 0.1% Tween 20 (Sigma-Aldrich, P1379) was used in all steps. Rabbit anti-UCH-L5 (Abcam, ab133508), rabbit anti-USP10 (Cell Signaling Technology, 8501), rabbit anti-USP25 antibody (Abcam, ab187156), and mouse anti-tubulin antibodies (Abcam, ab59680), respectively, were used for UCH-L5, USP10, USP25, and tubulin detection. Rabbit anti-CD9 (Cell Signaling Technology, 13174) was used for detection of the CD9 exosome marker.

After incubation with appropriate horseradish peroxidase-labeled secondary antibodies and washing membranes in TBS supplemented with 0.1% Tween 20, proteins were detected using enhanced chemiluminescence reagents (GE Healthcare, RPN2209 or Thermo Fisher Scientific, 34094). Images were visualized on medical X-Ray blue film (AGFA NV, XDAOG).

### 2.9 Silver nitrate staining of VDF membranes

Activated membranes were washed in distilled water and stained in staining solution prepared from 2% sodium citrate, 0.8% ferrous sulfate (FeSO_4_ · 7 H_2_O), 0.2% silver nitrate in water for 1 min. Then the membranes were rinsed with water and air-dried.

### 2.10 Semi-quantitative and quantitative analyses of DUBs transcription

Total RNA isolated from the infected or uninfected THP-1 cells using Allprep DNA/RNA MicroKit (Qiagen, 80004) was used for reverse transcription reaction (QuantiTect Reverse Transcription kit, Qiagen, 205311). The cDNA obtained was used as a template in subsequent polymerase chain reaction (PCR) reactions. Transcription of UCH-L5, USP10, and USP25 genes was analyzed by PCR using appropriate primers (**Supplementary Table 1**) and subsequent agarose gel electrophoresis. Transcription of GAPDH was used as a positive control. Intensities of resulting bands were analyzed using iBright Analysis software (Thermo Fisher Scientific, v5.0.1). The unpaired *t*-test in GraphPad Prism v6 software was used for statistical analysis.

Quantitative analysis using RT-qPCR for the transcription of genes of interest was performed using cDNA (described above), appropriate KiCqStart SYBR Green primers (**Supplementary Table 1**), and a QuantiTect SYBR Green PCR Kit (Qiagen, 204143). For qPCR and the data analysis, the 7500 Fast Real Time-PCR System (Applied Biosystems, 4377355) was used. Gene-specific amplification was confirmed by a single peak in the melting curve analysis. All gene transcription data are presented as the transcription relative to the GAPDH reference gene. The unpaired *t*-test in GraphPad Prism v6 software was used for statistical analysis.

### 2.11 Isolation of exosomes derived from THP-1

Exosomes derived from the uninfected or infected THP-1 macrophages were isolated by the ultracentrifugation method. Cell culture supernatants with secreted exosomes were collected 60 min post-infection. The samples were sequentially centrifuged first at 200 × g for 5 min, then at 5,000 × g for 10 min, and finally at 10,000 × g for 30 min, always at 4°C, to remove intact cells, cellular debris, and bacteria respectively. The supernatants were filtered through a 0.45 µm syringe filter (TPP, 99745). The samples were then ultracentrifuged at 100,000 × g for 70 min at 4°C. The supernatants were removed, the pelleted exosomes were washed with ice-cold PBS, and additional centrifugation was carried out at 100,000 × g for 70 min at 4°C. The exosomes were finally resuspended in PBS containing an EDTA-free protease inhibitor cocktail (Roche, 11836170001).

### 2.12 Imaging exosomes

For electron microscopy, the exosomes were visualized by negative staining. Parlodion-carbon-coated grids were floated on the top of a 10 µL drop of sample for 5 min. The grids were then stained by 2% uranyl acetate, for 2 × 30 s and dried. The grids were visualized with a JEOL JEM-2100 Plus electron microscope operated at 200 kV.

### 2.13 Nanoparticle tracking analysis of exosomes

The size and concentration of exosomes were assessed by nanoparticle tracking analysis (NTA) using a NanoSight NS300 analyzer (Malvern Instruments, United Kingdom) equipped with a green laser (532 nm), sCMOS camera, automatic injection pump system, and NTA 3.4 software. Before NTA measurements, isolated vesicles were diluted in 0.1 µm filtered PBS to achieve a concentration of approximately 1.0 × 10^8^ particles/mL. Five 60-second videos were recorded for each sample, with the camera level set to 16. The following settings were used for analysis: detection threshold 5, automatic blur size, automatic maximum track length, and automatic maximum expected particle size.

## 3 Results

### 3.1 Expression level of particular DUBs is not changed in THP-1 cells after *Francisella* infection

**Figure 1:**
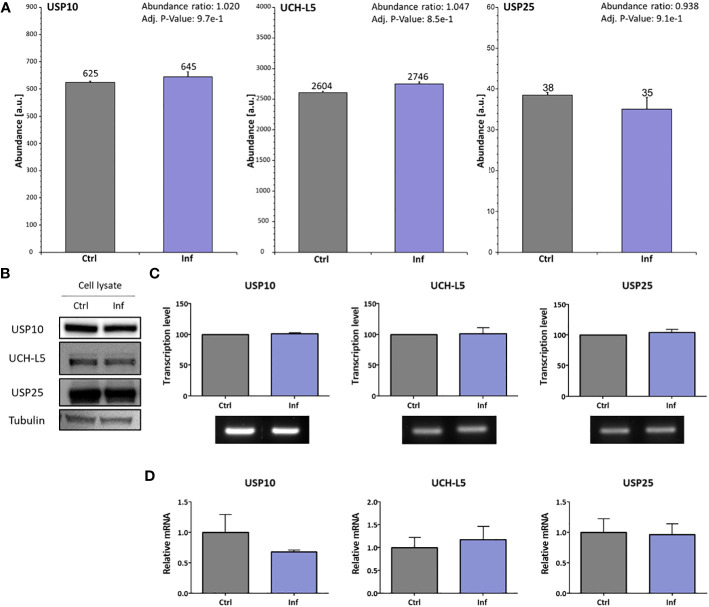
*F*. *tularensis* does not change the expression of selected DUBs in infected THP-1 macrophages. THP-1 macrophages were infected for 60 min with *F*. *tularensis*, after which the cell lysates were analyzed by nanoLC-MS using the TMT quantitation method. The abundances showed no significant changes in any of the tested DUB profiles. The MS abundances graphs for USP10, UCH-L5, and USP25 show no significant differences **(A)**. Western blot was used to verify the MS data **(B)**. The transcription level of USP10, UCH-L5, and USP25 was verified using RT-PCR **(C)**. Simultaneously, the RT-qPCR approach was used for the verification of RT-PCR results **(D)**. All data are based on three independent biological replicates. An unpaired *t*-test was used to establish statistical significance. Ctrl – uninfected, Inf – infected.

To study whether *Francisella* can manipulate the host deubiquitination system, we analyzed the expression level of selected DUBs in THP-1 macrophages after *Francisella* infection. In advance, the cell cytotoxicity at 10 and 60 min after infection was measured and no toxicity was found (**Supplementary Figure 1**). Next, we analyzed whole proteome changes after infection using TMT isobaric labeling quantitative proteomic method with a focus on DUBs (**Supplementary Table 2**). We found that the expression level of detected DUB proteins is not affected in THP-1 cells after *F. tularensis* subsp. *holarctica* FSC200 infection. We specifically focused on three enzymes reported to be involved in the immune response to bacterial infections: USP10, UCH-L5, and USP25 (**Figure 1A**). Using this proteomic approach, as well as Western blot immunoassays, we showed unchanged expression on the protein level for USP10, UCH-L5, and USP25 in THP-1 cell lysates after infection (**Figure 1B**). To complete our findings that the expression level of these three DUBs is not affected after infection, we performed an analysis of expression on the transcriptional level using RT-PCR (**Figure 1C**) and RT-qPCR (**Figure 1D**) methods. We confirmed that expression of USP10, UCH-L5, and USP25 is not affected on transcriptional level at 60 min after *F. tularensis* infection.

### 3.2 Overall activity of DUBs is altered in THP-1 cells after *Francisella* infection

**Figure 2:**
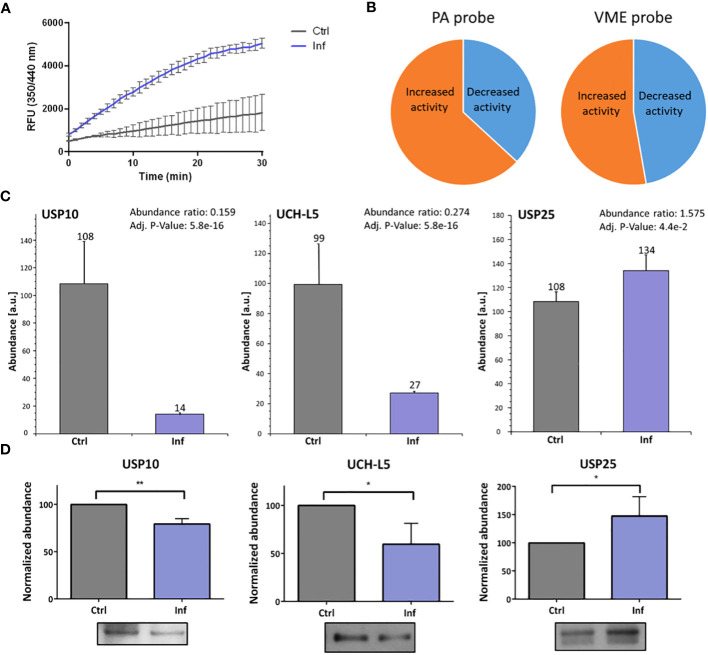
*F*. *tularensis* infection changes the DUBs’ activities in THP-1 macrophages. DUB activity assay was performed to detect DUB activity in THP-1 cell lysate at 60 min after infection. The results show changed DUB activity in infected cell lysates compared to the uninfected control. Upon infection, the overall activity of DUBs was increased **(A)**. The nanoLC-MS analysis of enriched DUBs upon reaction with HA-labeled probes confirmed changes in activity of all isolated DUBs after 60 min of infection. The pie charts illustrate the proportional changes in DUB activity **(B)**. The MS data confirmed the changes of activity in the cases of USP10 and UCH-L5 (both decreased) and of USP25 (increased) 60 min after infection **(C)**. Western blot analysis and comparison of bands densities were used for MS data evaluation **(D)**. The results of three biological replicates are shown. An unpaired *t*-test was used to establish statistical significance (*P< 0.05; **P< 0.01). Ctrl – uninfected, Inf – infected.

To analyze whether the host DUBs activity is changed during *Francisella* infection in human THP-1 cells, we performed DUBs activity assay at 10 and 60 min post-infection. We revealed that the activity of DUBs is not changed 10 min after infection (**Supplementary Tables 3**, **4**). On the other hand, we observed a significant increase in DUBs activity at 60 min after infection when compared to the uninfected control (**Figure 2A**). This finding led us to the question as to which DUBs are affected during infection. We used two ubiquitin-specific HA-tagged probes to mark active DUBs in the infected or uninfected THP-1 cell lysates. The cell-free lysates were obtained 60 min after infection and immunoprecipitations of the HA-Ub-PA and HA-Ub-VME-labeled DUBs were carried out. All experiments were done in triplicates. Samples were analyzed using high-performance liquid chromatography in combination with tandem mass spectrometry (HPLC-MS/MS) (**Supplementary Tables 5**, **6**). The results showed overall changes in the DUB cell profile (**Figure 2B**). We identified several DUBs showing significantly altered levels of expression when compared to uninfected control samples. In many cases, both affinity probes used may capture the same DUBs, however the capture efficiency may strongly differ among the probes. Thus the obtained LC-MS results (**Supplementary Tables 5**, **6**) have to be viewed critically and based on statistical significance of the measured abundance ratios, USP10 and UCH-L5 were unambiguously identified as changed DUBs in Ub-PA immunopurification. An interesting case is USP25 that may be involved in ubiquitination of TRAF3/TRAF6 (20), because TRAF3/TRAF6 ubiquitination is subverted during *Francisella* infection (11). LC-MS results of USP25 at 60 min were ambiguous and statistical evaluation provided marginally significant values. That is why an additional Western blot analysis for both affinity probes were done in order to shed light on the USP25 abundance change (**Supplementary Figure 2**) and the obtained corresponding results showed an increased abundance of USP25 in infected cells for both probes which was in agreement with the LC-MS results of the Ub-VME probe. USP10 and UCH-L5 showed then a decreased activity while USP25 showed an increased activity at 60 min after infection (**Figure 2C**), which was confirmed by a Western blot analysis (**Figure 2D**).

### 3.3 USP25, USP10, and UCH-L5 showed increased amounts in the exosomes after *Francisella* infection

**Figure 3:**
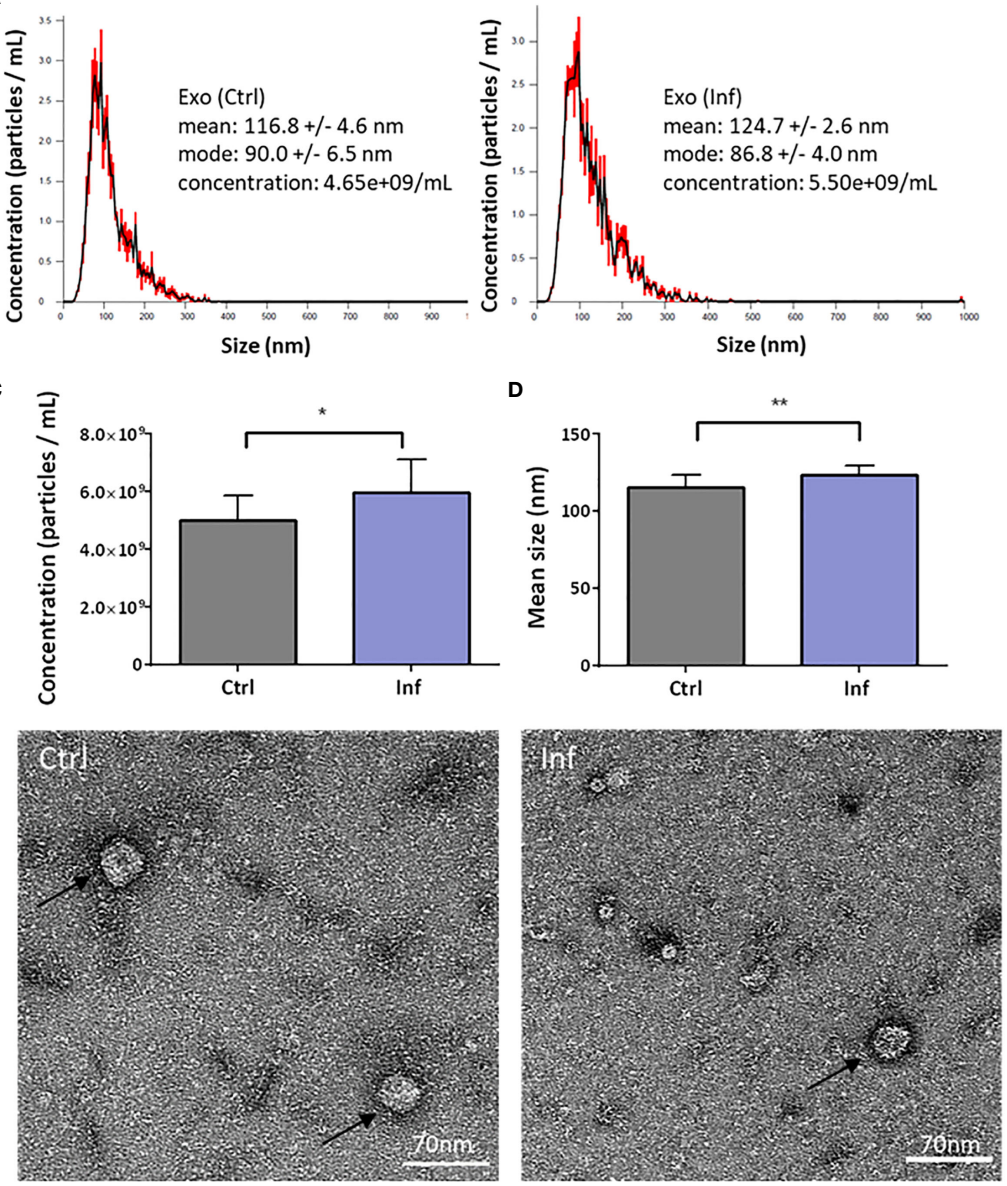
Characterization of the THP-1 exosomes. The exosomes were isolated 60 min after *Francisella* infection from cell culture supernatants using ultracentrifugation. The nanoparticle tracking analysis shows the size and concentration distribution of exosomes from uninfected versus infected THP-1 macrophages **(A, B)**. Representative graphs from three biological replicates are shown. Exosome particles concentration was increased after infection **(C)**, together with their size **(D)**. Exosomes (black arrows) were visualized by transmission electron microscopy **(E)**. An unpaired *t*-test was used to establish statistical significance (*P< 0.05; **P< 0.01). Ctrl – uninfected, Inf – infected.

In further analysis, we focused on THP-1-derived exosomes. We investigated whether the morphology of exosomes is affected or whether DUBs composition in exosomes is changed after infection. First, we characterized the size and quantity of exosomes produced by uninfected or infected THP-1 macrophages using NanoSite technology (**Figures 3A, B**). We showed that infected THP-1 cells produced significantly greater amounts of exosomes than did control (uninfected) samples (**Figure 3C**). We also observed significant differences in the size of exosomes that were produced by infected THP-1 cells when compared to the uninfected control (**Figure 3D**). The morphology of exosomes was visualized using electron microscopy (**Figure 3E**).

**Figure 4:**
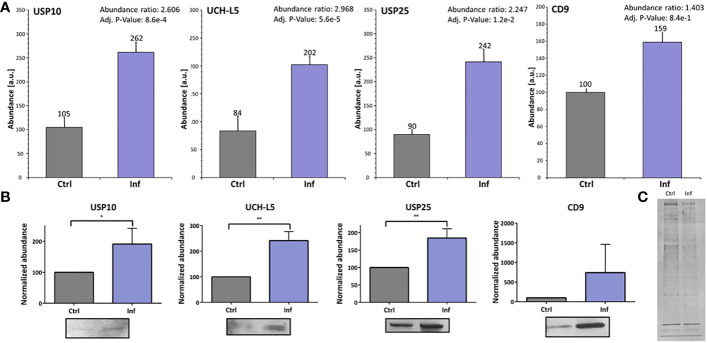
*Francisella* infection led to increased amounts of USP10, UCH-L5, and USP25 in THP-1 exosomes. The nanoLC-MS analysis of isolated exosomes showed significantly increased amounts of USP10, UCH-L5, and USP25 enzymes, as well as the exosome marker CD9 after 60 min of infection **(A)**. Representative Western blot analysis with comparison of bands densities were used to verify MS results **(B)**. As a loading control, silver nitrate staining of PVDF membrane was used **(C)**. The results of three biological replicates are shown and an unpaired t-test was used to establish statistical significance (*P< 0.05; **P< 0.01). Ctrl – uninfected, Inf – infected.

Next, we investigated whether the presence of individual DUBs is modulated after infection in the exosomes derived from THP-1 cells. We analyzed protein amount profiles of exosomes using the LFQ method to identify changes as a result of infection (**Supplementary Table 7**). We revealed that USP10, UCH-L5, and USP25 exhibited higher concentrations in exosomes after infection compared to uninfected control (**Figure 4A**). Increased amounts of these three particular enzymes in exosomes were confirmed using Western blot analysis and appropriate antibodies (**Figure 4B**). As a marker of exosomes, we choose CD9 (21). Interestingly, the CD9 marker showed increased abundance in exosomes isolated from the infected cells when compared to the uninfected control as confirmed by the MS approach as well as by Western blot analysis. This suggests that *Francisella* infection favors production of the CD9^+^ fraction of exosomes (**Figures 4A, B**; **Supplementary Table 7**). The abundance of CD9 marker was different between exosomes from infected and uninfected cells, thus the membrane silver nitrate staining was used as a loading control (**Figure 4C**).

## 4 Discussion

In the present work, we aimed to uncover the possible role of host deubiquitinases in the early phase of *Francisella* infection. The DUBs are important in various cell processes as enzymes counteracting ubiquitination. DUBs could play a role in delaying *Francisella* recognition and postponing immune system activation. *F. tularensis* Schu S4, the subspecies most virulent for both mice and humans, suppresses all pro-inflammatory responses for at least 72 h after infection (22). Decreased amount of K63-linked polyubiquitination and thus inhibition in the assembly of TRAF6 and TRAF3 complexes also have been observed at the first 60 min post-infection by *F. tularensis* LVS in bone marrow-derived macrophages (11), as have been essential changes in phosphorylation through the first 60 min in *F. tularensis* FSC200-infected bone marrow-derived dendritic cells (23) and therefore we focused on the early phase of infection.

Human PMA-derived THP-1 macrophages were used as host cells for infection with *F. tularensis* subsp. *holarctica* FSC200. While focusing on the early phase of *Francisella* infection and its intervention with DUB enzymes activation in human macrophages alongside DUBs composition changes in THP-1-produced exosomes, we proved a statistically significant difference in total DUB activity between infected and uninfected cells. Using label-free proteomics of specifically entrapped active DUBs, we identified decreased amounts of active USP10 and UCH-L5 in macrophages 60 min post-infection. On the contrary, the amount of active DUB USP25 was increased in the cells 60 min post-infection.

USP10, a cysteine protease, mediates the hydrolysis of peptide bonds between Ub and the targeted protein. It has been shown to be involved in various cellular processes, including regulation of cellular proteins, ubiquitin recycling, DNA damage response, and acting as a tumor suppressor (24). This enzyme is believed to play an essential role in regulating activity of the cystic fibrosis transmembrane conductance regulator (CFTR) and thus it is important in cystic fibrosis disease (25). *Pseudomonas aeruginosa* is known to produce Cif toxin that regulates CFTR deubiquitination by inhibition of USP10 (26), showing that pathogenic bacteria may regulate DUBs to prosper themselves. USP10 is also implicated in the regulation of autophagy through deubiquitination of beclin-1 (BECN1) (27, 28) and LC3b proteins (29). Deubiquitination of LC3b by USP10 causes an increased expression level of LC3b and concurrently induction of autophagy (29). In the proteomic data obtained from the TMT-labeled experiment, we found a decreased expression level of LC3b protein 60 min post-infection (PRIDE DOI: 10.6019/PXD043492) together with decreased activity of the USP10 enzyme (**Figures 2C, D**). We suggest that *Francisella* could control autophagy through regulating USP10 activity. *Francisella* is known to re-enter autophagosomes after its release into the cytoplasm at 12 h post-infection (30, 31). This means that later on the autophagy process can be controlled by the bacterium to enhance *Francisella*’s prosperity (32, 33). USP10 also deubiquitinates TRAF6 after formation of the complex with TRAF family member-associated NF-κB activator and monocyte chemotactic protein-1-induced protein 1, which negatively regulates genotoxic stress or IL-1β-mediated NF-κB activation (34). Considering the host–pathogen interaction, *Francisella* could modulate activity of the USP10 enzyme to suppress not only the NF-κB signaling pathway but the autophagy machinery as well.

The deubiquitinating enzyme UCH-L5 is associated with 26S proteasome, where it removes distal Ub moieties from polyubiquitinated proteins (35), and it is specific to K48-linked polyUb chains (36). UCH-L5 has a wide range of functions, interacting with multiple protein complexes to affect various cellular processes, including cooperation with the proteasome (37) and transcription factors (38). Roles of UCH-L5 in activation of NLRP3 inflammasome (39) was recently described. UCH-L5’s involvement in inflammasome activation has been found in several different pathogens (39–41). Increased expression of UCH-L5 enzyme leading to the activation of NLRP3 has been observed in macrophages infected by *Salmonella enterica* serovar Typhimurium. This led to activation of caspase-1 and subsequent cell pyroptosis and release of the bacteria into the extracellular space (40). *Francisella* dsDNA can be sensed by AIM2 inflammasome, which leads to caspase-1 activation and IL-1β release in murine macrophages (42, 43). It seems that *F. tularensis* FSC200 affects the activity of UCH-L5 in the first hour of infection in the opposite direction. The reduced activity of UCH-L5 might result in suppressing activation of NLRP3 inflammasome in the early phase of infection. In the later phases of infection, however, it was found that the NLRP3 reduces NF-κB and MAPK signaling, thereby promoting *Francisella’*s replication inside infected macrophages (44).

USP25, a ubiquitin-specific protease, plays a role, besides others, in bacterial infection (45–47). USP25 has been found to be a negative regulator of the NF-κB signaling pathway and thus to affect pro-inflammatory response (47, 48). Moreover, USP25 is upregulated by lipopolysaccharide (LPS) and thus ubiquitination of histone acetyltransferase HBO1 is suppressed, resulting in altered gene transcription (49). USP25 can hydrolyze both K48- and K63-linked polyUb chains. USP25 has been shown to be a negative regulator of pro-inflammatory response that is activated through IL-17 receptor, where TRAF5 and TRAF6 are deubiquitinated at K63-linked polyUb chains (ordinarily ubiquitinated by Act1 E3 ubiquitin ligase), thus leading to disruption of the NF-κB signaling pathway (20). USP25 is involved in the immune signaling pathway activated by toll-like receptors and LPS, where USP25 deubiquitinates K48-linked polyUb chains of TRAF3. This deubiquitination prevents TRAF3 from degrading and subsequently leads to the activation of interferon regulatory factor 3 transcription and thus type I interferon production (45). Putzova et al. (11) have shown that *F. tularensis* inhibits K63-linked polyubiquitination of TRAF3 and TRAF6 during infection, but more detailed study is greatly needed. Here, we reveal another piece of the puzzle to elucidate the deubiquitination machinery during *Francisella* infection.

In analyzing the proteome of exosomes produced by infected or uninfected cells, we found several changes in protein composition in exosomes upon infection. These include exosome markers, e.g. CD9 (**Supplementary Table 7**). Exosomes from infected cells show a higher level of CD9, which could indicate an attempt by infected cells to contact and warn neighboring cells of infection, similar to what was shown in study of 50. Further, we focused on DUBs enrichment in exosomes produced by *Francisella*-infected THP-1 cells. Exosomes produced by cells that had been infected by intracellular pathogens, such as *M. tuberculosis, Mycobacterium bovis, Salmonella typhimurium*, or *Toxoplasma gondii*, contain pathogen-associated molecular patterns and are able to stimulate a pro-inflammatory response (51). Biogenesis of endosomes, including those containing intraluminal vesicles, and subsequent release of exosomes are strongly affected by endosomal sorting complex required for transport machinery and ubiquitinated proteins (52). Whereas deubiquitination processes are ongoing during the final formation of exosomes, many polyubiquitinated non-integral membrane proteins have been found in exosomes (52). Exosomes derived from *M. tuberculosis-*infected cells contain mycobacterial proteins (53) that are ubiquitinated by macrophages, examples being GroES and HspX (54). *S. enterica* serovar Typhimurium-infected THP-1 cells were shown to produce exosomes with higher abundance of deubiquitinating enzyme OTUB1, thereby indicating the involvement of DUBs in bacterial infection (55). In our study, we found increased concentration and size of exosomes 60 min after infection by *Francisella* (**Figures 3C, D**). We next identified greater amounts of UCH-L5, USP10, and USP25 in THP-1 derived exosomes after *F. tularensis* FSC200 infection (**Figure 4**). Although the ubiquitination and deubiquitination processes in host–pathogen interactions are undoubtedly indispensable, the reason for and mechanism of ubiquitination of bacterial proteins in host’s exosomes after infection need to be better understood. Our findings could bring novel insight into the mechanism of *F. tularensis* infection and host–pathogen interaction, but further studies devoted to understanding the molecular mechanisms of how *F. tularensis* influences host infection to its advantage are much needed.

## Data availability statement

The datasets presented in this study can be found in online repositories. The names of the repository/repositories and accession number(s) can be found in the article/**Supplementary Material**.

## Ethics statement

Ethical approval was not required for the studies on humans in accordance with the local legislation and institutional requirements because only commercially available established cell lines were used.

## Author contributions

VV, PR, KH, PS, and PP conceived and designed the experiments. VV, KH, JH, RH, PS, and PP performed the experiments. VV, PR, KH, JH, RH, PS, and PP analyzed the data. VV, PP, PS, KH, PR, and JS wrote the paper. All authors contributed to the article and approved the submitted version.
