# Supplementary material for: Modified activities of macrophages’ deubiquitinating enzymes after Francisella infection
Source: Front Immunol. 2023 Sep 29;14:1252827. doi: 10.3389/fimmu.2023.1252827 (PMC10570801; doi:10.3389/fimmu.2023.1252827)
Supplement: Supplementary file 1 [file DataSheet_1.docx]

Supplementary Material

Activities of macrophages’ deubiquitinating enzymes are altered after Francisella infection

Vera Vozandychova^1*^, Pavel Rehulka^1^, Kamil Hercik^1,2^, Petra Spidlova^1^, Pavla Pavlik^1^_,_ Jaroslav Hanus^3^, Romana Hadravova^2^, and Jiri Stulik^1^

*** Correspondence:** Vera Vozandychova: vera.vozandychova@unob.cz

# Supplementary Figures and Tables

## Supplementary Figures

Supplementary Figure 1 The F. tularensis infection is not cytotoxic for human macrophages. THP-1 macrophages were infected by bacteria at MOI of 500:1 and compared with the uninfected control. The LDH cytotoxicity assay of culture supernatants from cells infected for 10 or 60 min was performed. The results are from three biological replicates. Ctrl – uninfected, Inf – infected.


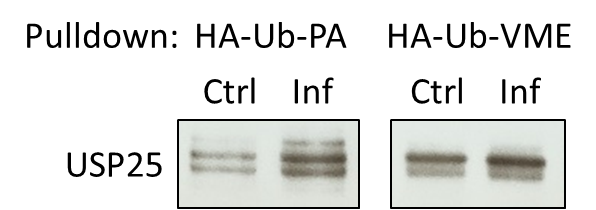


Supplementary Figure 2 Western blot analysis of USP25 enzyme. Western blot analysis of the proteins isolated from macrophages using HA-Ub-PA and HA-Ub-VME affinity probes at 60 min after infection with *F. tularensis* and antibody detection of USP25. Ctrl – uninfected, Inf – infected.

## Supplementary Tables

Supplementary Table 1 List of KiCqStart SYBR Green primers (Sigma-Aldrich, KSPQ12012)

| **Oligo Name** | **Lenght** | **MW** | **Tm°** | **GC %** | **Sequence (5'-3')** |
| --- | --- | --- | --- | --- | --- |
| Forward Human 1 GAPDH | 18 | 5558 | 62.4 | 50 | TCGGAGTCAACGGATTTG |
| Reverse Human 1 GAPDH | 23 | 6964 | 59.1 | 39.1 | CAACAATATCCACTTTACCAGAG |
| Forward Human 1 UCHL5 | 22 | 6799 | 58.7 | 40.9 | CCACTAGTAGAAAAGGCAAAAG |
| Reverse Human 1 UCHL5 | 20 | 6218 | 59 | 40 | TATGTGCAGAAGCAGAAATG |
| Forward Human 1 USP10 | 20 | 6114 | 53.7 | 45 | GACTCCTGACAGTTAACAAG |
| Reverse Human 1 USP10 | 21 | 6329 | 61 | 42.8 | CAACATTTCCTCATGAAGTCC |
| Forward Human 1 USP25 | 20 | 6150 | 57.4 | 40 | AATGTGATTGATCTCACTGG |
| Reverse Human 1 USP25 | 21 | 6310 | 58.2 | 42.8 | TCATCAGTTATTCCAGTCTCC |

Supplementary Table 2 Identified DUBs in cell lysates after 60 min infection by F. tularensis subsp. holarctica FSC200 by proteomic analysis with using TMT isobaric labeling

| **Accession** | **Description** | **Coverage [%]** | **# Unique Peptides** | **MW [kDa]** | **calc. pI** | **Abundance Ratio: (inf) / (ctrl)** | **Abundance Ratio Adj. P-Value: (inf) / (ctrl)** |
| --- | --- | --- | --- | --- | --- | --- | --- |
| Q86UV5 | Ubiquitin carboxyl-terminal hydrolase 48 | 4 | 4 | 119 | 6.05 | 0.902 | 5.70E-01 |
| P51784 | Ubiquitin carboxyl-terminal hydrolase 11 | 5 | 2 | 109.7 | 5.45 | 0.908 | 6.20E-01 |
| O94966 | Ubiquitin carboxyl-terminal hydrolase 19 | 7 | 8 | 145.6 | 6.28 | 0.954 | 7.74E-01 |
| Q13107 | Ubiquitin carboxyl-terminal hydrolase 4 | 11 | 6 | 108.5 | 5.71 | 1.05 | 8.14E-01 |
| Q96K76 | Ubiquitin carboxyl-terminal hydrolase 47 | 8 | 10 | 157.2 | 5.08 | 1.046 | 8.52E-01 |
| Q9Y5K5 | Ubiquitin carboxyl-terminal hydrolase isozyme L5 | 32 | 10 | 37.6 | 5.33 | 1.044 | 8.52E-01 |
| Q9UPU5 | Ubiquitin carboxyl-terminal hydrolase 24 | 6 | 15 | 294.2 | 6.14 | 0.964 | 8.77E-01 |
| P45974 | Ubiquitin carboxyl-terminal hydrolase 5 | 12 | 10 | 95.7 | 5.03 | 1.037 | 9.00E-01 |
| Q96JH7 | Deubiquitinating protein VCPIP1 | 12 | 13 | 134.2 | 7.2 | 1.037 | 9.08E-01 |
| Q9UHP3 | Ubiquitin carboxyl-terminal hydrolase 25 | 2 | 2 | 122.1 | 5.34 | 0.95 | 9.11E-01 |
| Q8NBR6 | Ubiquitin carboxyl-terminal hydrolase MINDY-2 | 4 | 2 | 67.1 | 4.51 | 1.065 | 9.16E-01 |
| Q9Y5T5 | Ubiquitin carboxyl-terminal hydrolase 16 | 3 | 2 | 93.5 | 6.93 | 1.087 | 9.16E-01 |
| Q96BN8 | Ubiquitin thioesterase otulin | 11 | 5 | 40.2 | 5.47 | 1.035 | 9.19E-01 |
| Q9Y4E8 | Ubiquitin carboxyl-terminal hydrolase 15 | 15 | 13 | 112.3 | 5.22 | 1.031 | 9.31E-01 |
| Q92995 | Ubiquitin carboxyl-terminal hydrolase 13 | 5 | 4 | 97.3 | 5.53 | 1.056 | 9.35E-01 |
| Q70CQ2 | Ubiquitin carboxyl-terminal hydrolase 34 | 1 | 4 | 404 | 5.82 | 1.051 | 9.59E-01 |
| Q93009 | Ubiquitin carboxyl-terminal hydrolase 7 | 20 | 21 | 128.2 | 5.55 | 1.022 | 9.59E-01 |
| P15374 | Ubiquitin carboxyl-terminal hydrolase isozyme L3 | 40 | 7 | 26.2 | 4.92 | 0.99 | 9.70E-01 |
| P54578 | Ubiquitin carboxyl-terminal hydrolase 14 | 37 | 15 | 56 | 5.3 | 1.018 | 9.70E-01 |
| Q9NUU6 | Inactive ubiquitin thioesterase OTULINL | 14 | 7 | 42.2 | 9.29 | 0.993 | 9.73E-01 |
| Q8N6M0 | Deubiquitinase OTUD6B | 10 | 3 | 33.8 | 60.5 | 1.017 | 9.76E-01 |
| P40818 | Ubiquitin carboxyl-terminal hydrolase 8 | 10 | 10 | 127.4 | 8.51 | 1.018 | 9.80E-01 |
| P46736 | Lys-63-specific deubiquitinase BRCC36 | 22 | 6 | 36 | 5.92 | 1.016 | 9.81E-01 |
| Q14694 | Ubiquitin carboxyl-terminal hydrolase 10 | 12 | 8 | 87.1 | 5.31 | 1.011 | 9.81E-01 |
| Q96FW1 | Ubiquitin thioesterase OTUB1 | 37 | 8 | 32.3 | 4.94 | 1.01 | 9.88E-01 |
| Q93008 | Probable ubiquitin carboxyl-terminal hydrolase FAF-X | 9 | 22 | 290.3 | 5.8 | 1.027 | 9.44E-01 |
| Q9P275 | Ubiquitin carboxyl-terminal hydrolase 36 | 4 | 5 | 122.8 | 9.7 | 1.005 | 9.96E-01 |

Supplementary Table 3 Identified DUBs in HA-Ub-PA probe enriched cell lysates after 10 min infection by *F. tularensis* subsp. *holarctica* FSC200 by LFQ proteomic analysis

| **Accession** | **Description** | **Coverage [%]** | **# Unique Peptides** | **MW [kDa]** | **calc. pI** | **Abundance Ratio: (inf, PA) / (ctrl, PA)** | **Abundance Ratio Adj. P-Value: (inf, PA) / (ctrl, PA)** |
| --- | --- | --- | --- | --- | --- | --- | --- |
| J3KR44 | Ubiquitin thioesterase OTUB1 | 51 | 10 | 31.4 | 5 | 0.819 | 5.15E-01 |
| Q5SZ59 | OTU domain containing 7B (Fragment) | 19 | 6 | 47.6 | 5.36 | 0.801 | 6.52E-01 |
| Q9UPU5 | Ubiquitin carboxyl-terminal hydrolase 24 | 22 | 46 | 294.2 | 6.14 | 0.894 | 8.81E-01 |
| Q9UHP3 | Ubiquitin carboxyl-terminal hydrolase 25 | 9 | 7 | 122.1 | 5.34 | 1.134 | 8.91E-01 |
| Q93008-1 | Isoform 2 of Probable ubiquitin carboxyl-terminal hydrolase FAF-X | 26 | 30 | 290.3 | 5.8 | 0.902 | 8.98E-01 |
| Q9Y5T5 | Ubiquitin carboxyl-terminal hydrolase 16 | 20 | 12 | 93.5 | 6.93 | 0.911 | 9.21E-01 |
| G5E9A6 | Ubiquitin carboxyl-terminal hydrolase 11 | 27 | 16 | 105 | 5.33 | 0.933 | 9.50E-01 |
| Q96JH7 | Deubiquitinating protein VCIP135 | 5 | 5 | 134.2 | 7.2 | 1.056 | 9.73E-01 |
| Q9NUU6 | Inactive ubiquitin thioesterase OTULINL | 10 | 4 | 42.2 | 9.29 | 1.061 | 9.73E-01 |
| Q70CQ2 | Ubiquitin carboxyl-terminal hydrolase 34 | 1 | 3 | 404 | 5.82 | 1.066 | 9.77E-01 |
| A0A087X0W9 | Deubiquitinase OTUD6B | 8 | 2 | 37.3 | 6.74 | 0.935 | 9.78E-01 |
| Q14694 | Ubiquitin carboxyl-terminal hydrolase 10 | 12 | 6 | 87.1 | 5.31 | 1.061 | 9.83E-01 |
| P45974 | Ubiquitin carboxyl-terminal hydrolase 5 | 41 | 29 | 95.7 | 5.03 | 1.033 | 9.83E-01 |
| P40818 | Ubiquitin carboxyl-terminal hydrolase 8 | 33 | 32 | 127.4 | 8.51 | 0.959 | 9.84E-01 |
| Q01804 | OTU domain-containing protein 4 | 14 | 11 | 124 | 6.71 | 0.967 | 9.90E-01 |
| P54578 | Ubiquitin carboxyl-terminal hydrolase 14 | 51 | 21 | 56 | 5.3 | 0.972 | 9.90E-01 |
| Q96K76-4 | Isoform 4 of Ubiquitin carboxyl-terminal hydrolase 47 | 41 | 48 | 154.6 | 5.06 | 1.008 | 9.93E-01 |
| O00507 | Probable ubiquitin carboxyl-terminal hydrolase FAF-Y | 12 | 1 | 290.9 | 5.86 | 0.973 | 9.93E-01 |
| Q9Y4E8 | Ubiquitin carboxyl-terminal hydrolase 15 | 55 | 48 | 112.3 | 5.22 | 0.978 | 9.93E-01 |
| Q13107 | Ubiquitin carboxyl-terminal hydrolase 4 | 47 | 32 | 108.5 | 5.71 | 0.978 | 9.93E-01 |
| P15374 | Ubiquitin carboxyl-terminal hydrolase isozyme L3 | 64 | 13 | 26.2 | 4.92 | 0.98 | 9.93E-01 |
| Q5LJA5 | Ubiquitin carboxyl-terminal hydrolase UCHL5 | 59 | 17 | 40.4 | 5.34 | 0.977 | 9.93E-01 |
| Q9Y6I4 | Ubiquitin carboxyl-terminal hydrolase 3 | 10 | 4 | 58.9 | 8.18 | 0.991 | 9.96E-01 |
| Q93009 | Ubiquitin carboxyl-terminal hydrolase 7 | 50 | 47 | 128.2 | 5.55 | 0.988 | 9.99E-01 |
| O94966-6 | Isoform 6 of Ubiquitin carboxyl-terminal hydrolase 19 | 30 | 23 | 151.8 | 6.42 | 0.992 | 1.00E+00 |

Supplementary Table 4 Identified DUBs in HA-Ub-VME probe enriched cell lysates after 10 min infection by *F. tularensis* subsp. *holarctica* FSC200 by LFQ proteomic analysis

| **Accession** | **Description** | **Coverage [%]** | **# Unique Peptides** | **MW [kDa]** | **calc. pI** | **Abundance Ratio: (inf, VME) / (ctrl, VME)** | **Abundance Ratio Adj. P-Value: (inf, VME) / (ctrl, VME)** |
| --- | --- | --- | --- | --- | --- | --- | --- |
| **Q9Y5K5** | **Ubiquitin carboxyl-terminal hydrolase isozyme L5** | **65** | **1** | **37.6** | **5.33** | **0.65** | **9.65E-05** |
| Q9NVE5 | Ubiquitin carboxyl-terminal hydrolase 40 | 4 | 4 | 140 | 5.67 | 0.789 | 1.38E-01 |
| P54578 | Ubiquitin carboxyl-terminal hydrolase 14 | 49 | 22 | 56 | 5.3 | 1.151 | 6.13E-01 |
| Q6GQQ9 | OTU domain-containing protein 7B | 17 | 10 | 92.5 | 6.71 | 0.881 | 7.01E-01 |
| Q14694 | Ubiquitin carboxyl-terminal hydrolase 10 | 47 | 26 | 87.1 | 5.31 | 0.881 | 7.01E-01 |
| Q9Y5T5 | Ubiquitin carboxyl-terminal hydrolase 16 | 28 | 17 | 93.5 | 6.93 | 0.893 | 7.82E-01 |
| Q96JH7 | Deubiquitinating protein VCIP135 | 7 | 7 | 134.2 | 7.2 | 1.108 | 8.48E-01 |
| Q93008-1 | Isoform 2 of Probable ubiquitin carboxyl-terminal hydrolase FAF-X | 29 | 65 | 290.3 | 5.8 | 0.91 | 8.84E-01 |
| Q92560 | Ubiquitin carboxyl-terminal hydrolase BAP1 | 13 | 7 | 80.3 | 6.84 | 0.913 | 8.85E-01 |
| Q9Y6I4 | Ubiquitin carboxyl-terminal hydrolase 3 | 42 | 17 | 58.9 | 8.18 | 0.913 | 8.86E-01 |
| Q5LJA5 | Ubiquitin carboxyl-terminal hydrolase UCHL5 | 60 | 1 | 40.4 | 5.34 | 0.922 | 9.28E-01 |
| Q9UPU5 | Ubiquitin carboxyl-terminal hydrolase 24 | 23 | 46 | 294.2 | 6.14 | 0.924 | 9.33E-01 |
| Q93009 | Ubiquitin carboxyl-terminal hydrolase 7 | 56 | 52 | 128.2 | 5.55 | 0.929 | 9.50E-01 |
| P45974 | Ubiquitin carboxyl-terminal hydrolase 5 | 38 | 29 | 95.7 | 5.03 | 1.074 | 9.50E-01 |
| Q96K76-4 | Isoform 4 of Ubiquitin carboxyl-terminal hydrolase 47 | 46 | 53 | 154.6 | 5.06 | 1.062 | 9.63E-01 |
| B4E3L3 | Ubiquitin carboxyl-terminal hydrolase 28 | 2 | 2 | 105.8 | 5.35 | 0.94 | 9.63E-01 |
| J3KR44 | Ubiquitin thioesterase OTUB1 | 13 | 3 | 31.4 | 5 | 1.062 | 9.63E-01 |
| Q86UV5 | Ubiquitin carboxyl-terminal hydrolase 48 | 10 | 8 | 119 | 6.05 | 0.944 | 9.64E-01 |
| Q01804 | OTU domain-containing protein 4 | 11 | 8 | 124 | 6.71 | 0.955 | 9.85E-01 |
| A0A087X0W9 | Deubiquitinase OTUD6B | 17 | 4 | 37.3 | 6.74 | 0.961 | 9.89E-01 |
| Q9UHP3 | Ubiquitin carboxyl-terminal hydrolase 25 | 37 | 32 | 122.1 | 5.34 | 0.978 | 9.89E-01 |
| K7EII4 | Ubiquitin carboxyl-terminal hydrolase 32 (Fragment) | 9 | 2 | 28.3 | 7.23 | 1.042 | 9.89E-01 |
| P40818 | Ubiquitin carboxyl-terminal hydrolase 8 | 31 | 6 | 127.4 | 8.51 | 1.033 | 9.89E-01 |
| Q9NQC7 | Ubiquitin carboxyl-terminal hydrolase CYLD | 11 | 10 | 107.2 | 5.59 | 0.971 | 9.89E-01 |
| G5E9A6 | Ubiquitin carboxyl-terminal hydrolase 11 | 28 | 17 | 105 | 5.33 | 1.036 | 9.89E-01 |
| Q9NUU6 | Inactive ubiquitin thioesterase OTULINL | 5 | 2 | 42.2 | 9.29 | 0.987 | 9.91E-01 |
| P15374 | Ubiquitin carboxyl-terminal hydrolase isozyme L3 | 55 | 12 | 26.2 | 4.92 | 1.015 | 9.91E-01 |
| O75604 | Ubiquitin carboxyl-terminal hydrolase 2 | 7 | 3 | 68 | 8.97 | 0.988 | 9.95E-01 |
| O94966-6 | Isoform 6 of Ubiquitin carboxyl-terminal hydrolase 19 | 32 | 25 | 151.8 | 6.42 | 1.012 | 9.96E-01 |
| Q70CQ2 | Ubiquitin carboxyl-terminal hydrolase 34 | 2 | 6 | 404 | 5.82 | 1.011 | 9.96E-01 |
| Q13107 | Ubiquitin carboxyl-terminal hydrolase 4 | 44 | 32 | 108.5 | 5.71 | 1.005 | 9.96E-01 |
| Q9Y4E8 | Ubiquitin carboxyl-terminal hydrolase 15 | 50 | 44 | 112.3 | 5.22 | 1.001 | 9.99E-01 |
| P40818-2 | Isoform 2 of Ubiquitin carboxyl-terminal hydrolase 8 | 29 | 1 | 115 | 8.46 |  |  |

Supplementary Table 5 Identified DUBs in HA-Ub-PA probe enriched cell lysates after 60 min infection by *F. tularensis* subsp. *holarctica* FSC200 by LFQ proteomic analysis

| **Accession** | **Description** | **Coverage [%]** | **# Unique Peptides** | **MW [kDa]** | **calc. pI** | **Abundance Ratio: (PA, inf) / (PA, ctrl)** | **Abundance Ratio Adj. P-Value: (PA, inf) / (PA, ctrl)** |
| --- | --- | --- | --- | --- | --- | --- | --- |
| **Q14694** | **Ubiquitin carboxyl-terminal hydrolase 10** | **27** | **17** | **87.1** | **5.31** | **0.159** | **5.76E-16** |
| **Q9Y5K5-3** | **Isoform 3 of Ubiquitin carboxyl-terminal hydrolase isozyme L5** | **69** | **21** | **37.5** | **5.4** | **0.274** | **5.76E-16** |
| **A0A087X0W9** | **Deubiquitinase OTUD6B** | **15** | **6** | **37.3** | **6.74** | **0.579** | **4.80E-03** |
| Q86UV5 | Ubiquitin carboxyl-terminal hydrolase 48 | 6 | 5 | 119 | 6.05 | 0.608 | 2.05E-02 |
| Q9UHP3 | Ubiquitin carboxyl-terminal hydrolase 25 | 23 | 21 | 122.1 | 5.34 | 0.608 | 2.35E-02 |
| Q9Y6I4 | Ubiquitin carboxyl-terminal hydrolase 3 | 9 | 5 | 58.9 | 8.18 | 0.621 | 3.20E-02 |
| J3KRR7 | Ubiquitin carboxyl-terminal hydrolase CYLD | 4 | 3 | 101.8 | 5.49 | 0.688 | 1.93E-01 |
| O75317 | Ubiquitin carboxyl-terminal hydrolase 12 | 6 | 2 | 42.8 | 6.6 | 0.7 | 3.19E-01 |
| Q92560 | Ubiquitin carboxyl-terminal hydrolase BAP1 | 13 | 9 | 80.3 | 6.84 | 0.747 | 6.21E-01 |
| Q9NVE5 | Ubiquitin carboxyl-terminal hydrolase 40 | 2 | 3 | 140 | 5.67 | 0.815 | 8.06E-01 |
| Q9NUU6 | Inactive ubiquitin thioesterase OTULINL | 6 | 3 | 42.2 | 9.29 | 0.875 | 9.32E-01 |
| J3QS30 | Ubiquitin carboxyl-terminal hydrolase 22 (Fragment) | 17 | 2 | 11.7 | 8.79 | 0.917 | 9.72E-01 |
| P51784 | Ubiquitin carboxyl-terminal hydrolase 11 | 18 | 14 | 109.7 | 5.45 | 0.973 | 9.81E-01 |
| E9PEW0 | Ubiquitin carboxyl-terminal hydrolase 36 | 12 | 6 | 60.3 | 9.52 | 1.034 | 9.13E-01 |
| Q9Y5T5 | Ubiquitin carboxyl-terminal hydrolase 16 | 22 | 14 | 93.5 | 6.93 | 1.035 | 9.02E-01 |
| P40818-2 | Isoform 2 of Ubiquitin carboxyl-terminal hydrolase 8 | 35 | 1 | 115 | 8.46 | 1.038 | 9.29E-01 |
| Q70CQ2 | Ubiquitin carboxyl-terminal hydrolase 34 | 1 | 4 | 404 | 5.82 | 1.078 | 9.01E-01 |
| P40818 | Ubiquitin carboxyl-terminal hydrolase 8 | 39 | 9 | 127.4 | 8.51 | 1.094 | 8.25E-01 |
| P45974 | Ubiquitin carboxyl-terminal hydrolase 5 | 34 | 28 | 95.7 | 5.03 | 1.097 | 8.13E-01 |
| Q13107 | Ubiquitin carboxyl-terminal hydrolase 4 | 36 | 37 | 108.5 | 5.71 | 1.11 | 8.06E-01 |
| Q9Y4E8 | Ubiquitin carboxyl-terminal hydrolase 15 | 44 | 41 | 112.3 | 5.22 | 1.112 | 8.06E-01 |
| Q9UPU5 | Ubiquitin carboxyl-terminal hydrolase 24 | 26 | 57 | 294.2 | 6.14 | 1.116 | 8.06E-01 |
| O94966-5 | Isoform 5 of Ubiquitin carboxyl-terminal hydrolase 19 | 22 | 22 | 155.9 | 6.38 | 1.119 | 8.06E-01 |
| Q96K76-4 | Isoform 4 of Ubiquitin carboxyl-terminal hydrolase 47 | 39 | 43 | 154.6 | 5.06 | 1.121 | 8.06E-01 |
| Q6GQQ9 | OTU domain-containing protein 7B | 12 | 9 | 92.5 | 6.71 | 1.122 | 7.90E-01 |
| Q93009 | Ubiquitin carboxyl-terminal hydrolase 7 | 48 | 56 | 128.2 | 5.55 | 1.123 | 8.00E-01 |
| B4E3L3 | Ubiquitin carboxyl-terminal hydrolase 28 | 3 | 2 | 105.8 | 5.35 | 1.161 | 8.00E-01 |
| P54578 | Ubiquitin carboxyl-terminal hydrolase 14 | 39 | 17 | 56 | 5.3 | 1.164 | 7.06E-01 |
| Q93008-1 | Isoform 2 of Probable ubiquitin carboxyl-terminal hydrolase FAF-X USP9X | 26 | 32 | 290.3 | 5.8 | 1.168 | 6.96E-01 |
| Q8NB14 | Ubiquitin carboxyl-terminal hydrolase 38 | 2 | 2 | 116.5 | 6.21 | 1.197 | 7.30E-01 |
| O75604 | Ubiquitin carboxyl-terminal hydrolase 2 | 12 | 5 | 68 | 8.97 | 1.215 | 5.14E-01 |
| Q96JH7 | Deubiquitinating protein VCIP135 | 9 | 8 | 134.2 | 7.2 | 1.278 | 3.47E-01 |
| P15374 | Ubiquitin carboxyl-terminal hydrolase isozyme L3 | 70 | 15 | 26.2 | 4.92 | 1.282 | 2.55E-01 |
| Q5VVQ6 | Ubiquitin thioesterase OTU1 | 10 | 3 | 38.3 | 6.11 | 1.305 | 3.15E-01 |
| **J3KR44** | **Ubiquitin thioesterase OTUB1** | **47** | **12** | **31.4** | **5** | **1.512** | **8.34E-03** |
| Q01804 | OTU domain-containing protein 4 | 3 | 3 | 124 | 6.71 | 1.517 | 2.66E-02 |
| Q9H9J4 | Ubiquitin carboxyl-terminal hydrolase 42 | 2 | 2 | 145.3 | 8.63 |  | 5.76E-16 |
| O00507 | Probable ubiquitin carboxyl-terminal hydrolase FAF-Y USP9Y | 11 | 1 | 290.9 | 5.86 |  | 5.76E-16 |

Supplementary Table 6 Identified DUBs in HA-Ub-VME probe enriched cell lysates after 60 min infection by *F. tularensis* subsp. *holarctica* FSC200 by LFQ proteomic analysis

| **Accession** | **Description** | **Coverage [%]** | **# Unique Peptides** | **MW [kDa]** | **calc. pI** | **Abundance Ratio: (VME, inf) / (VME, ctrl)** | **Abundance Ratio Adj. P-Value: (VME, inf) / (VME, ctrl)** |
| --- | --- | --- | --- | --- | --- | --- | --- |
| Q01804 | OTU domain-containing protein 4 | 3 | 3 | 124 | 6.71 | 0.01 | 6.79E-16 |
| J3KR44 | Ubiquitin thioesterase OTUB1 | 32 | 7 | 31.4 | 5 | 0.598 | 5.37E-02 |
| Q96JH7 | Deubiquitinating protein VCIP135 | 6 | 6 | 134.2 | 7.2 | 0.809 | 8.89E-01 |
| Q6GQQ9 | OTU domain-containing protein 7B | 12 | 8 | 92.5 | 6.71 | 0.816 | 8.99E-01 |
| K7EQL6 | Ubiquitin carboxyl-terminal hydrolase 32 | 2 | 2 | 143.8 | 6.64 | 0.867 | 9.49E-01 |
| Q13107 | Ubiquitin carboxyl-terminal hydrolase 4 | 33 | 34 | 108.5 | 5.71 | 0.868 | 9.49E-01 |
| P45974 | Ubiquitin carboxyl-terminal hydrolase 5 | 33 | 27 | 95.7 | 5.03 | 0.904 | 9.69E-01 |
| B4E3L3 | Ubiquitin carboxyl-terminal hydrolase 28 | 3 | 2 | 105.8 | 5.35 | 0.905 | 9.69E-01 |
| P40818 | Ubiquitin carboxyl-terminal hydrolase 8 | 36 | 9 | 127.4 | 8.51 | 0.928 | 9.94E-01 |
| P51784 | Ubiquitin carboxyl-terminal hydrolase 11 | 18 | 14 | 109.7 | 5.45 | 0.946 | 9.97E-01 |
| P40818-2 | Isoform 2 of Ubiquitin carboxyl-terminal hydrolase 8 | 32 | 1 | 115 | 8.46 | 0.952 | 9.97E-01 |
| Q9NVE5 | Ubiquitin carboxyl-terminal hydrolase 40 | 2 | 3 | 140 | 5.67 | 0.965 | 9.99E-01 |
| Q9Y6I4 | Ubiquitin carboxyl-terminal hydrolase 3 | 20 | 12 | 58.9 | 8.18 | 0.97 | 9.97E-01 |
| Q9NUU6 | Inactive ubiquitin thioesterase OTULINL | 4 | 2 | 42.2 | 9.29 | 0.979 | 9.97E-01 |
| O75604 | Ubiquitin carboxyl-terminal hydrolase 2 | 12 | 5 | 68 | 8.97 | 0.983 | 9.97E-01 |
| P15374 | Ubiquitin carboxyl-terminal hydrolase isozyme L3 | 70 | 15 | 26.2 | 4.92 | 0.988 | 9.97E-01 |
| O94966-5 | Isoform 5 of Ubiquitin carboxyl-terminal hydrolase 19 | 21 | 20 | 155.9 | 6.38 | 0.99 | 9.97E-01 |
| Q9UPU5 | Ubiquitin carboxyl-terminal hydrolase 24 | 24 | 54 | 294.2 | 6.14 | 0.991 | 9.97E-01 |
| Q9Y4E8 | Ubiquitin carboxyl-terminal hydrolase 15 | 42 | 38 | 112.3 | 5.22 | 1.01 | 9.89E-01 |
| Q9Y5T5 | Ubiquitin carboxyl-terminal hydrolase 16 | 25 | 16 | 93.5 | 6.93 | 1.019 | 9.78E-01 |
| Q93008-1 | Isoform 2 of Probable ubiquitin carboxyl-terminal hydrolase FAF-X USP9X | 25 | 31 | 290.3 | 5.8 | 1.034 | 9.69E-01 |
| A0A075B784 | Ubiquitin carboxyl-terminal hydrolase 36 | 8 | 7 | 104.3 | 9.76 | 1.036 | 9.69E-01 |
| O00507 | Probable ubiquitin carboxyl-terminal hydrolase FAF-Y USP9Y | 11 | 1 | 290.9 | 5.86 | 1.052 | 9.67E-01 |
| A0A087X0W9 | Deubiquitinase OTUD6B | 20 | 6 | 37.3 | 6.74 | 1.068 | 9.55E-01 |
| Q96K76-4 | Isoform 4 of Ubiquitin carboxyl-terminal hydrolase 47 | 38 | 42 | 154.6 | 5.06 | 1.089 | 9.35E-01 |
| Q70CQ2 | Ubiquitin carboxyl-terminal hydrolase 34 | 2 | 7 | 404 | 5.82 | 1.093 | 9.27E-01 |
| Q93009 | Ubiquitin carboxyl-terminal hydrolase 7 | 48 | 51 | 128.2 | 5.55 | 1.103 | 9.21E-01 |
| Q9Y5K5-3 | Isoform 3 of Ubiquitin carboxyl-terminal hydrolase isozyme L5 | 69 | 20 | 37.5 | 5.4 | 1.164 | 8.54E-01 |
| Q9NQC7 | Ubiquitin carboxyl-terminal hydrolase CYLD | 8 | 7 | 107.2 | 5.59 | 1.166 | 8.53E-01 |
| Q92560 | Ubiquitin carboxyl-terminal hydrolase BAP1 | 18 | 12 | 80.3 | 6.84 | 1.188 | 8.02E-01 |
| Q86UV5 | Ubiquitin carboxyl-terminal hydrolase 48 | 11 | 9 | 119 | 6.05 | 1.263 | 6.24E-01 |
| P54578 | Ubiquitin carboxyl-terminal hydrolase 14 | 37 | 17 | 56 | 5.3 | 1.308 | 4.92E-01 |
| Q9UPT9 | Ubiquitin carboxyl-terminal hydrolase 22 | 4 | 2 | 59.9 | 8.05 | 1.312 | 4.83E-01 |
| Q14694 | Ubiquitin carboxyl-terminal hydrolase 10 | 31 | 20 | 87.1 | 5.31 | 1.405 | 2.42E-01 |
| H7BZK6 | Ubiquitin carboxyl-terminal hydrolase 46 | 6 | 2 | 39.5 | 7.36 | 1.519 | 8.30E-02 |
| **Q9UHP3** | **Ubiquitin carboxyl-terminal hydrolase 25** | **36** | **32** | **122.1** | **5.34** | **1.575** | **4.44E-02** |
| Q9H9J4 | Ubiquitin carboxyl-terminal hydrolase 42 | 2 | 2 | 145.3 | 8.63 |  |  |

Supplementary Table 7 Identified human proteins in exosome fraction after 60 min infection by *F. tularensis* subsp. *holarctica* FSC200 by LFQ proteomic analysis

| **Accession** | **Description** | **# Unique Peptides** | **Abundance Ratio: Inf / Ctrl** | **Abundance Ratio Adj. P-Value: Inf / Ctrl** | **Protein type** |
| --- | --- | --- | --- | --- | --- |
| Q96I24 | Far upstream element-binding protein 3 | 2 | 5.137 | 7.27E-12 | Other |
| Q6S8J3 | POTE ankyrin domain family member E | 1 | 3.394 | 2.42E-06 | Other |
| **Q9Y5K5** | **Ubiquitin carboxyl-terminal hydrolase isozyme L5** | **8** | **2.968** | **5.64E-05** | **DUB** |
| **Q14694** | **Ubiquitin carboxyl-terminal hydrolase 10** | **4** | **2.606** | **8.64E-04** | **DUB** |
| A0A590UJJ4 | Voltage-gated potassium channel subunit beta-2 | 1 | 2.538 | 1.34E-03 | Other |
| **Q9UHP3** | **Ubiquitin carboxyl-terminal hydrolase 25** | **6** | **2.247** | **1.16E-02** | **DUB** |
| F8VWK8 | Tetraspanin (Fragment) | 4 | 2.229 | 1.24E-02 | Marker |
| P53999 | Activated RNA polymerase II transcriptional coactivator p15 | 4 | 2.135 | 2.41E-02 | Enzyme |
| O14828 | Secretory carrier-associated membrane protein 3 | 4 | 2.083 | 3.34E-02 | Marker |
| A0A4W8VX11 | Pericentriolar material 1 protein | 3 | 1.973 | 6.82E-02 | Other |
| P21926 | CD9 antigen | 3 | 1.403 | 8.41E-01 | Marker |
| P08133 | Annexin A6 | 25 | 0.807 | 8.41E-01 | Marker |
| P50995 | Annexin A11 | 12 | 0.77 | 7.50E-01 | Marker |
| P09525 | Annexin A4 | 6 | 0.743 | 6.28E-01 | Marker |
| P20073 | Annexin A7 | 4 | 0.727 | 5.88E-01 | Marker |
| E7EQB2 | Lactotransferrin (Fragment) | 2 | 0.578 | 7.65E-02 | Transporter |
| E9PEX6 | Dihydrolipoyl dehydrogenase | 2 | 0.572 | 6.82E-02 | Enzyme |
| P01857 | Immunoglobulin heavy constant gamma 1 | 2 | 0.543 | 3.34E-02 | Immune response |
| P30101 | Protein disulfide-isomerase A3 | 13 | 0.519 | 1.74E-02 | Enzyme |
| P23141 | Liver carboxylesterase 1 | 3 | 0.515 | 1.59E-02 | Enzyme |
| P04083 | Annexin A1 OS=Homo sapiens | 5 | 0.51 | 1.36E-02 | Marker |
| P23284 | Peptidyl-prolyl cis-trans isomerase B | 4 | 0.484 | 6.03E-03 | Enzyme |
| P27797 | Calreticulin | 7 | 0.411 | 3.07E-04 | Chaperone |
| P13667 | Protein disulfide-isomerase A4 | 5 | 0.39 | 9.87E-05 | Enzyme |
| K7ELL7 | Glucosidase 2 subunit beta | 4 | 0.367 | 2.54E-05 | Enzyme |
| P40926 | Malate dehydrogenase, mitochondrial | 5 | 0.361 | 1.90E-05 | Enzyme |
